# Frey syndrome prevalence after partial parotidectomy

**DOI:** 10.1016/S1808-8694(15)30042-2

**Published:** 2015-10-19

**Authors:** Regiane Cristina Santos, Jose Francisco de Salles Chagas, Thiago Freire Pinto Bezerra, Jose Eli Baptistella, Marcos Alberto Pagani, Alexandre Rocha Melo

**Affiliations:** a MD, 3rd year resident; b Head and Neck Surgeon, PhD; Head and Neck Preceptor; c MD; 3rd year resident; d MD; Otolaryngologist - Head of the Otolaryngology department; e Head and Neck Surgeon, Otolaryngologist - Head and Neck Preceptor; f MD - 2nd year resident Santa Casa De Misericordia De Limeira

**Keywords:** Frey syndrome, Gustatory Sweating, Partial Parotidectomy, Sternocleidomastoid Muscle Flap

## Abstract

**Introduction:**

Frey syndrome is a sequela observed after parotidectomy and the reported incidence varies enormously in the literature. Diagnosis is evaluated by presence of the classic triad of gustatory sweating, heating and flushing while feeding and documented by Minor starch-iodine test.

**Aim:**

to evaluate the incidence of this syndrome in patients submitted to partial parotidectomy at Centro Otorrinolaringológico de Limeira, from 1994 to 2004, including presence of signs and symptoms and the surgical technique.

**Material and method:**

fourteen patients undergoing partial parotidectomy with sternocleidomastoid muscle flap answered a questionnaire and were classified as positive or negative by Minor starch-iodine test in a clinical retrospective study.

**Results:**

21% of the patients presented symptoms and positive iodine test.

**Conclusion:**

only the patients presenting clinical symptoms had a positive test and the adopted surgical technique was efficient due to low incidence of the syndrome.

## INTRODUCTION

Frey Syndrome (FS) or gustatory sudoresis, although present in the literature since 1757, in Duphenix report, it was introduced and characterized in a series of cases, as it is known today, only since 1853, by Ballanger^1^. It is characterized by facial hyperemia, heat and sudoresis, especially in the parotid and malar regions at chewing. Historically described in the literature as a post-parotidectomy complication with variable prevalence, it depends on factors such as post-operative interval, surgical technique and method used for sudoresis evaluation. Symptoms usually crop up six weeks after surgery, time the injured nerve takes to regenerate, but they may come later, there are literature reports of 14 years^2^, and they may be triggered by any type of gustatory stimulus. The classic syndrome triad is hyperemia, heat and sweating in the pre-mandibular region and the mandible angle area^3^. Diagnosis is hinted according to patient's complaint of sweating related or not to mastication, that is, involuntary^3^; and confirmed by the iodine starch coloring method^4^. Although Lucie Frey did not describe the first case of facial gustatory hyperemia and sudoresis, she really deserved having the syndrome named after her, for not only she correctly described the symptoms, but she also provided the correct insight on the relevance of the autonomic innervation of the skin and parotid gland in 1923. When she accurately described the role the auriculotemporal nerve has in the Frey syndrome, she provided the missing link between gustatory food stimulation from the one hand and the facial sudoresis on the other. However, it was André Thomas who correctly explained the physiopathology when he postulated the aberrant regeneration theory^5^. The most accepted theory in this syndrome pathogenesis is the aberrant regeneration theory, in which the auriculotemporal nerve fibers are damaged during surgery and, in the regeneration process the parotid parasympathetic fibers join the subcutaneous sweat glands sympathetic fibers. Therefore, at a salivary reflex during mastication, besides saliva production, there is a stimulus towards sweat and local vasodilatation, happening with hyperemia^6^.

## OBJETIVE

The goal of this paper is to assess the incidence of FS in patients who undergo partial parotidectomy with filling of the parotid fossa with a pedicled flap of sternocleidomastoid muscle at the Limeira ENT center, correlated to the symptoms.

## MATERIALS AND METHODS

This study was developed at the Limeira ENT Center, after due authorization from the Santa Casa de Misericordia de Limeira Ethics Committee. We studied 14 patients who underwent partial parotidectomy. They all signed an informed consent. They all answered a questionnaire where they stated post operative time and complaints of symptoms such as hyperemia and sweating during a meal, and underwent the iodine starch coloring test.

The iodine and starch test comprises brushing 3% ethanol solution with iodine on the skin. After drying, we spread starch powder over it and offered the patient a sialagogue, lemon juice drops. After that, we observed the presence or absence of sweating and blue-black coloring reaction on the area studied, thus identifying the presence of gustatory syndrome.

Exclusion criteria were: patients who underwent total parotidectomy; less then 6 weeks of post operative time; previous history of facial trauma and non-parotid head and neck previous surgeries.

The analysis as to whether or not the syndrome was present was carried out through clinical interview and iodine test, through a qualitative analysis, and the patients were divided into positive and negative. We did not assess the affected area size.

The surgical technique used for partial parotidectomy followed these steps: 1) anti-sepsis cleaning with iodine solution; 2) pre-auricular vertical skin incision extending 2cm downwards, posterior to the mandible angle; 3) lifting the anterior skin flap going beyond the parotid lesion in about 2cm; and posteriorly, until exposure of the sternocleidomastoid muscle and external jugular vein exposure; 4) dissection of the parotid tissue present in the sternocleidomastoid muscle and exposure of the digastric muscle posterior belly; 5) dissecting the facial nerve trunk and its branches until beyond the parotid lesion in about 2cm; 6) resection of the parotid lesion, with 1cm of safety margin; 7) rotation of the sternocleidomastoid muscle pedicled flap enough to cover the surgical wound; 8) flap fixation to the parotid gland remaining parenchyma with polyglycolic acid suture wire separated stitches3-0; 9) hemostasis review; 10) flushing the surgical field with 0.09% saline solution; 11) leaving a laminar drainage line in the surgical wound and fixed to the skin; 12) suture of subcutaneous tissue and skin with 3-0 multifilament polyglycolic acid wire and 4-0 monofilament nylon wire, respectively.

## RESULTS

The analysis of these 14 patients who underwent partial parotidectomy showed that the post-operative time varied between 6 weeks and 10 years. The iodine test was positive in 3 cases (21) and they were the same patients that, in answering the questionnaire, mentioned facial sweating, heat and flush during mastication.

In the negative test patients (79%), none presented signs and symptoms compatible to FS, showing a correlation between the clinical complaint and the iodine test positiveness.

## DISCUSSION

Since it was first described in 1853, many papers have been published regarding FS, describing its etiology, clinical aspects, incidence and treatment. We are still waiting to see a consensus about its incidence, for in the publications we studied the incidence rate varied from 2 to 80%^7^.

Historically, parotid benign tumor surgeries has evolved from a simple enucleating of the parotid nodule up to total parotidectomy, with or without facial nerve preservation, depending on tumor size, location, previous surgeries and histopathological origin^8^.

The surgical technique adopted may be associated to a number of complications such as hematoma, salivary fistulas, facial nerve hypoesthesia and those secondary to the auriculotemporal nerve, a branch of the glossopharyngeal nerve and responsible for parasympathetic innervation of the parotid, and sympathetic innervation of the facial sweat glands. These autonomic fibers are cholinergic and, as a consequence, patients in the post operative of parotid region surgeries may develop FS, due to it section and anomalous re-innervation^9^. This may be related to the surgical technique employed in parotidectomies, because we see no report of FS in enucleating procedures. In cases of superficial parotidectomies, there are publications describing 4.4% rates, however such fact may be related to a lack of direct interviewing the patient, because the study is only descriptive^7^, ^9^. Other reports, in which questionnaires and iodine tests were used to diagnose FS, show results varying from 36% to 50%^10^, ^11^, ^12^. Luna-Ortiz, Sansón-RioFrio and Mosqueda-Taylor stated that there is no difference in FS incidence in patients who undergo total or superficial parotidectomy^13^.

Classically, superficial parotidectomy comprises the resection of all the superficial gland lobe together with the disease being treated, in order to reduce the possibility of local recurrence; besides there being a risk, even if mild, of multicentricity of the treated disease, as is the case of pleomorphic adenomas. These factors were studied by Wen, Chen and Wang through histopathology cross sections of 25 parotid gland pleomorphic adenomas and they concluded that tumor extension beyond its capsule varied from 0.09 to 0.285mm and that, in two multicentric cases, the lesions were found together, bound by a single tumor capsule^14^. Therefore, the surgical technique may be altered, maintaining a healthy gland tissue margin of 1 cm, thus, avoiding greater handling of the facial nerve and glossopharyngeal nerve branches, and, consequently, less incidence of FS, as we saw in our study, in which we had a prevalence of only 21%.

The parotid gland has sympathetic innervation from the cervical plexus; and parasympathetic innervation from the glossopharyngeal and the auriculotemporal nerves, which may be damaged during surgery, even in small interventions. Therefore, any approach to the parotid gland where we may have glandular tissue trauma and, consequently trauma to the innervation, may cause FS when we have the regeneration of damaged nerves^15^.

In this paper we found FS positiveness in 21% of the patients who underwent partial parotidectomy, in agreement with literature results that indicate partial parotidectomy as proper treatment for benign parotid tumors^14^, ^15^, and corroborating the already reported surgical approaches to reduce FS, such as rotating the sternocleidomastoid muscle flap^16^.

The choice for a sternocleidomastoid muscle flap is based on its upper and lower vascularization, which facilitates its viability maintenance. In our cases, we used the upper based sternocleidomastoid muscle rotation, although the literature reports on a case of a bi-pedicled sternocleidomastoid muscle flap, subcutaneous fat graft, use of temporoparietal fascia to cover the operating wound. When one decides to use subcutaneous fat graft or temporoparietal fascia, he/she will require longer surgical time, besides the disadvantages of needing a second incision, and possibly a 30% resorption of the grafted tissue. Despite being a feasible promise, Alloderm^17^ grafts, processed allogenic grafts, are yet to be assessed in the long term for parotid use and may increase the cost of surgery depending on the amount used. Although the use of bi-pedicled flaps is being recently advocated in order to avoid the need to resect the muscle, which may cause ischemic atrophy or denervation, and thus enhance the cosmetic result18, this was not seen in our patients, even with the use of single pedicle grafts.

Our study showed an important 100% correlation between clinical complaint and FS diagnosed through the iodine test, proving the validity of the interview data in the routine post operative assessment of parotidectomies for its diagnosis.

## CONCLUSION

The surgical technique employed in our service was efficient in treating the gland pathology and presented low FS incidence. Only symptomatic patients presented positive iodine tests.
